# Surgical Technique and Implementation of Total Minimally Invasive (Laparo-Thoracoscopic) Ivor Lewis Esophagectomy for Cancer

**DOI:** 10.3390/cancers16193281

**Published:** 2024-09-26

**Authors:** Francesco Puccetti, Silvia Battaglia, Agnese Carresi, Lorenzo Cinelli, Stefano Turi, Ugo Elmore, Riccardo Rosati, the OSR CCeR Collaborative Group

**Affiliations:** 1 Department of Gastrointestinal Surgery, IRCCS San Raffaele Scientific Institute, 20132 Milan, Italy; battaglia.silvia@hsr.it (S.B.); carresi.agnese@hsr.it (A.C.); cinelli.lorenzo@hsr.it (L.C.); elmore.ugo@hsr.it (U.E.); rosati.riccardo@hsr.it (R.R.); 2 School of Medicine, Vita-Salute San Raffaele University, 20132 Milan, Italy; 3 Department of Anesthesia and Intensive Care, IRCCS San Raffaele Scientific Institute, 20132 Milan, Italy; turi.stefano@hsr.it; 4 Department of Rehabilitation and Functional Recovery, IRCCS San Raffaele Scientific Institute, 20132 Milan, Italy; centro.esofago@hsr.it; 5 Clinical Nutrition, IRCCS San Raffaele Scientific Institute, 20132 Milan, Italy

**Keywords:** esophagectomy, esophageal cancer, minimally invasive surgery, operative description

## Abstract

**Simple Summary:**

The minimally invasive approach has been applied to esophageal cancer surgery since the last decade of the 20th century to reduce the notoriously high levels of postoperative morbidity and respiratory complications. In the last twenty years, due to all the encouraging demonstrations of clinical feasibility, safety, and oncological effectiveness, minimally invasive esophagectomy has gradually incorporated multiple elements of technical heterogeneity, creating individualized approaches for indications for surgery and operative techniques. This article combines all the procedural details and interdisciplinary requirements from a high-volume experience in esophageal cancer care and aims to provide a comprehensive description of the total minimally invasive (laparo-thoracoscopic) procedure for the two-field Ivor Lewis esophagectomy.

**Abstract:**

Background/Objectives: Esophagectomy represents a major oncological operation due to the surgical involvement of both the abdominal and thoracic cavities. The minimally invasive technique has been developed to minimize the operative impact on patients undergoing esophageal resections, often presenting with nutritional deterioration and poor functional reserves. Methods: The present article provides an illustrative description of the total minimally invasive (laparo-thoracoscopic) Ivor Lewis esophagectomy for cancer integrated with complementary components of perioperative clinical management. This standardized surgical technique of two-field esophagectomy (i.e., laparoscopy and thoracoscopy) was depicted based on the experience of a tertiary center for esophageal cancer care with more than 1500 cases operated on, and in accordance with the SUPER reporting guidelines. Results and conclusions: The accomplishment of the following descriptive and illustrative content allowed the development of remarks on the strengths and possible flaws of this specific procedure, providing a measurable opportunity to absorb technical details of the most widespread surgical resection for esophageal cancer worldwide.

## 1. Introduction

Esophageal cancer (EC) is globally the seventh leading cause of cancer death, with an estimated incidence and mortality rate of 5.1 new cases and 4.5 deaths per 100,000, respectively [1]. The poor prognosis and the high morbidity in EC care led to the establishment of the multimodal approach combining different therapeutics (i.e., chemo or chemoradiotherapy and more recently immunotherapy) with esophageal resections, which remain the predominant curative component. In the 1940s, Ivor Lewis (1895–1982) developed a two-field (i.e., laparotomy and right-sided thoracotomy) esophagectomy for the dissection of neoplastic lesions of the middle third of the esophagus [2]. The operative technique was first presented in Lewis’s Hunterian Lecture at the Royal College of Surgeons of England (10 January 1946) and subsequently established as one of the most reliable esophageal resections.

The earliest programs of minimally invasive esophageal resections date back to the 1990s, with the introduction of laparoscopic transhiatal esophagectomies [3,4]. In 1993, Peracchia et al. described the first cases of thoracoscopic three-field esophagectomies, with intrathoracic esophageal dissection and hand-sewn cervical anastomosis [5,6]. In 1999, Watson et al. reported the total minimally invasive approach for Ivor Lewis esophagectomy, which consecutively combined laparoscopy and right-sided thoracoscopy with intrathoracic anastomosis [7]. Afterward, minimally invasive esophagectomy incorporated a wide variety of intrathoracic anastomotic techniques, such as the hand-sewn end-to-side anastomosis with full-thickness interrupted or oversewn running suture [7,8], the mechanical (linear-stapled) side-to-side anastomosis [9], and the mechanical (circular-stapled) end-to-side anastomosis [10,11,12]. Over time, the minimally invasive platform has embedded several technological developments (i.e., imaging magnification, improving energy devices, intraoperative fluorescence, or robotic assistance), contributing to an exponential increase in procedural variations and infrastructural resources, which were flexibly applied according to the individual surgical practices and availability. The present report aims to provide a comprehensive and stepwise illustration of the surgical stages and technical details of total minimally invasive (laparo-thoracoscopic) Ivor Lewis esophagectomy (MILE-lt) for cancer, with curative intent.

## 2. Materials and Methods

The surgical procedure of MILE-lt was described in compliance with the “Surgical technique rePorting chEcklist and standaRd” (SUPER) reporting guidelines (Supplementary Material: SUPER Checklist) [13]. The present surgical technique has been developed and reported at the Department of Gastrointestinal Surgery at the IRCCS San Raffaele Scientific Institute, representing the tertiary center for esophageal cancer with the highest hospital volume in Italy. In particular, Prof. Riccardo Rosati (Chief of the Gastrointestinal Unit and senior author of this article) was formerly trained under Prof. Alberto Peracchia’s mentorship and then conceived the current MILE-lt technique, involving a laparo-thoracoscopic esophageal resection for cancer with curative intent. At the moment of the present report, the institutional experience counts a series of 1500 esophagectomies over the last 20 years, and MILE-lt represents the resection type of choice as well as the vast majority of all operations (68.1%). Throughout the next part of this dissertation, the authors will expose the strengths and possible limitations of the reported technique in terms of surgical indications, clinical benefits, and infrastructural and professional requirements.

### Indications and Technical Limitations

The two-field Ivor Lewis esophagectomy was originally introduced as a surgical procedure for neoplastic diseases growing from the middle third of the intrathoracic esophagus to the esophagogastric junction, pursuing the underlying belief of a superior exposure and control during the thoracic stage [14]. Promoters of the right-sided approach to the thoracic esophagus advocated superior advantages in accomplishing extended mediastinal lymphadenectomy, longer esophageal safety margin, and preservation of gastric reservoir [15]. Over time, driving criteria for adequate surgical resections of junctional cancers were generally developed based on local infiltration (i.e., according to the Siewert classification) or the expected locoregional (either abdominal or thoracic) spread [16,17]. On the other hand, the minimally invasive technique historically conveyed multiple advantages to esophageal cancer surgery, leading to improved clinical outcomes and largely spreading over high-volume centers worldwide [18]. For these reasons, indications for MILE-lt for esophageal cancer include all cases presenting with tumor locations between the distal esophagus (i.e., middle or lower third) and the esophagogastric junction (i.e., Siewert type I or II). Conversely, possible limitations to MILE-lt may be associated with specific disease characteristics that could reduce the treatment effectiveness, such as a tumor location out of the above-mentioned range (i.e., cervical, proximal thoracic, or gastric levels, including Siewert type III), and findings of enlarged dimensions of bulky primary tumors or extensive retroperitoneal lymph node involvement. Also, patient susceptibility to pneumoperitoneum could require a conversion to the open approach, while neoadjuvant therapy does not represent a limitation for Ivor Lewis esophagectomy or the minimally invasive technique.

## 3. Multidisciplinary Complements

### 3.1. Multidisciplinary Management

Esophageal cancer care involves a wide range of multidisciplinary professionals who tailor multimodal strategies and specific treatments based on individual clinical stage and tumor and patient characteristics.

After histological confirmation and the prior clinical assessment of eligibility for surgery, esophageal cancer cases are routinely discussed within a multidisciplinary tumor board, which completes the clinical staging and establishes the subsequent therapeutic strategy. Conforming to the international guidelines, all patients presenting with locally advanced esophageal cancer (>cT1b, Nx, M0) are submitted to neoadjuvant therapy (i.e., chemo or chemoradiotherapy) in accordance with disease and patient features [19]. Further to the multidisciplinary agreement, clinical and administrative support is provided by the Navigator Nurse, who subsequently drives patients and monitors their tolerance and functional fluctuations throughout the whole clinical pathway.

Prehabilitation has been recently developed to enhance patient fitness before multiple fields of surgery and particularly showed potential in the preoperative optimization of esophageal cancer patients. Physiotherapy is a predominant component of prehabilitation, which can be remotely assisted, with the purpose of respiratory muscle reinforcement and cardio-pulmonary function improvement prior to esophagectomy. Nutritional assessment should be mandatory from the time of the diagnosis in order to address the presenting deterioration and identify possible areas of improvement. In cases of complete dysphagia or severe malnourishment (Nutritional Risk Score screening > 3) [20], feeding jejunostomy should be placed and enteral nutrition administered to ensure patients have optimal support during the neoadjuvant treatments. Preoperative work-up systematically includes patients’ and caregivers’ education, optimization of personal medications and comorbidities, and anesthesiologic evaluation of functional reserves and the required level of postoperative care.

Concerning the perioperative management of esophagectomy, standardized clinical protocols have definitely spread throughout high-volume centers, achieving lower rates of operative stress and improved clinical results [21]. Our institution established a specific ERAS-based perioperative protocol, which has been implemented since 2012 and includes procedure-specific components, as previously described [22].

### 3.2. Anesthetic Considerations

Intraoperative anesthetic management embraces the multidimensional monitoring and correction of all the possible interactions between MILE-lt and patient vital functions.

Respiratory management begins with double-lumen intubation or bronchial blocker placement, which allows switching to single-lung ventilation during specific moments of the thoracic stage. Evidence from retrospective studies supported the clinical benefits and feasibility of two-lung ventilation (TLV) during thoracoscopic esophagectomy, which can be facilitated by the effect of intrathoracic carbon dioxide insufflation [23]. According to the literature, TLV can potentially lead to reduced pulmonary complications and preserved respiratory functions after surgery, although this specific evidence still needs to be confirmed by randomized controlled trials (RCTs) [21].

Fluid balance over esophageal resections should aim to maintain adequate ranges of mean arterial pressure (MAP) during the extended perioperative period, which contributes to steadier gastric conduit perfusion and minimized pulmonary dysfunctions due to the fluid balance restrictions. Recently, a multicenter Japanese RCT demonstrated the interactions between goal-directed therapy and stroke volume variation and the resulting effectiveness in reducing morbidity and mortality after esophagectomy [24].

Eventually, the conception and establishment of standardized perioperative protocols, including anesthetic management of either hemodynamic deteriorations or pain control, demonstrated measurable clinical advantages after esophageal resections [25]. In particular, the introduction of the thoracic paravertebral catheter reported the highest efficacy under both parameters, demonstrating a non-inferior analgesic effect (i.e., pain control) along with less postoperative hypotension rates (i.e., impact on hemodynamics) in our institutional series [26].

## 4. Operative Technique

### 4.1. Setting and Positioning

**Figure 1:**
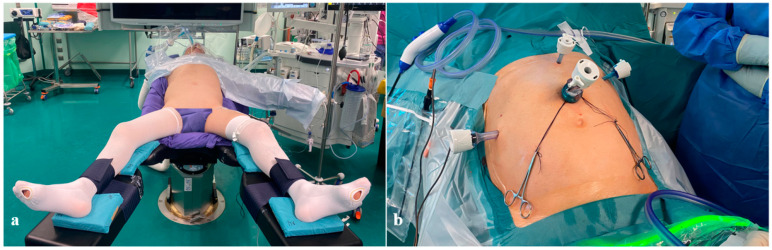
Settings of the abdominal stage. (**a**) Patient positioning; (**b**) trocar deployment: 5–12 mm optical trocar above the umbilicus; 5–12 mm right paraumbilical trocar; 5 mm upper-medial trocar; 5 mm upper-left trocar.

The patient initially lies supine, with legs apart and a 30–45-degree anti-Trendelenburg tilt. The patient is positioned as depicted in Figure 1a and supplied with central and arterial lines.

The surgical team includes one surgeon (standing in the middle), two assistants, and a scrub nurse on the surgeon’s right and left sides, respectively. The surgical equipment and instrumentations for the following surgical technique has been described in the Supplementary Material (Supplementary Material: Surgical instrument equipment). The pneumoperitoneum is generally performed through the open technique, and the height of the first midline incision should be tailored to the patient’s physical characteristics and BMI. Raising the level of the Hasson trocar could better provide an optimal view in higher-BMI patients, allowing a comprehensive visualization of celiac lymph nodes, including the most posterior stations. Abdominal trocar deployment is depicted in Figure 1b.

**Figure 2:**
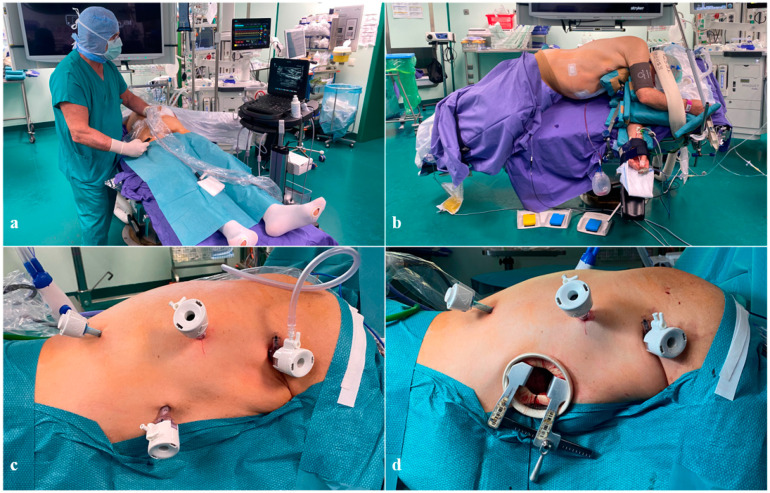
Settings of the thoracic stage. (**a**) Ultrasound-guided injection of ICG at the bilateral groin lymph nodes; (**b**) patient positioning; (**c**) trocar deployment: 5–12 mm optical trocar at the scapula’s inferior angle; 5–12 mm trocar at the apex of the posterior axillary line; 5–12 mm trocar at the fifth intercostal space and the anterior axillary line intersection; 5 mm trocar at the lowest intercostal space along the paravertebral line; (**d**) trocar deployment including the minithoracotomy.

After the abdominal stage and the inguinal intranodal ICG injection (Figure 2a), the patient is turned to a left-side lateral position, as shown in Figure 2b. An axillary roll is placed under the lower chest, and bracket supports are bilaterally clamped to the table, holding the sternum anteriorly and pelvis posteriorly. The patient’s legs are separated by a pillow and wrapped in secure straps. The left leg is flexed at 90 degrees, and the right one is straight. Similarly, the left arm is straight, while the right one is folded in front of the head. The operative table is turned in a “semi-prone” position to a 45° tilt of the operative table. The semi-prone position enables the deployment of the intrathoracic fluids in the inferior part of the thoracic cavity without compromising the view of the operative field. Also, reaching the semi-prone position through bed rotation represents a safety choice, which facilitates the conversion to open surgery in case of unexpected intraoperative complications. Thoracic trocar deployment is illustrated in Figure 2c,d. 

### 4.2. Abdominal Stage

After deploying the abdominal trocars, a smooth dissection of the lateral peritoneal attachments of the duodenum (Kocher maneuver) is performed to enhance the antro-pyloric mobilization of the stomach. A full-thickness pyloromyotomy with pyloroplasty is performed to prevent postoperative delayed gastric conduit emptying (DGCE). Although other centers do not routinely include the Kocher maneuver and pyloromyotomy in their standard practice, the authors strongly recommend both for the following reasons: (i) minimal surgical burden and operative time increase, (ii) prevention of limited transposition of the gastric conduit, (iii) prevention of postoperative DGCE, and (iv) inability to digitally assess pyloric patency or perform muscular stretching (digitoclasia). The dissection of the lesser omentum exposes the celiac and suprapancreatic retroperitoneum, starting from the “crow’s foot”, where the distal branches of the anterior and posterior vagal trunks innervate the antro-pyloric region near the incisura angularis. This anatomical benchmark guides the lesser omentum dissection, sparing the right gastric vessels and marking the beginning of the gastric conduit shaping. The celiac lymphadenectomy requires accuracy and follows the adventitial plan of the hepatic artery from the hepatoduodenal ligament (st. 12a), along the common hepatic artery and hepatoportal space (st. 8a, 8p), and down the celiac axis (st. 9). The dissection at the left gastric vein and artery origin includes nodes around the left gastric artery and the lesser curvature (st. 7, 3). Proximal and distal suprapancreatic excision (st. 11p, 11d) should preserve the splenic artery integrity, while the dissection of right and left cardiac nodes (st. 1, 2) completes the en bloc subdiaphragmatic lymphadenectomy. Gastrolysis along the greater curvature is achieved by dissecting the gastrocolic (with the preservation of the right gastroepiploic artery and arcade) and gastrosplenic ligament (with the interruption of short gastric and left gastroepiploic vessels).

After gastrolysis, indocyanine green (ICG) angiography highlights gastroepiploic arcade integrity and visceral perfusion, essential for the gastric conduit creation. A colopexy is normally performed at this stage, suturing the dissected part of the greater omentum to the left hemi-diaphragm; this allows the distal transverse colon to remain stable in the abdomen, preventing the migration of the omentum and colon into the thoracic cavity, which normally has an incidence of up to 10% postoperatively. A stapling suture starts from the “crow’s foot” and goes parallel to the greater curvature, creating a conduit (40–50 mm wide) up to the gastric fundus and in continuity with the esophagogastric junction. The right crus is partially opened to allow easy transition of the gastric tube from the abdominal to the thoracic cavity. Eventually, the dissection of mediastinal adhesions allows the opening of the right pleura and the transhiatal placement of a Jackson–Pratt suction drain in the right pleural cavity. In our experience, this transhiatal pleural drain represents a valid alternative to the chest tube, with effective draining and significantly reduced intercostal pain.

### 4.3. Thoracic Stage

After laparoscopy and before turning the patient position, an ultrasound-guided injection of ICG bilaterally at the groin lymph nodes allows the lymphatic spread of the fluorescent tracer (lymphography) with the clear visualization of the thoracic duct (TD), which appears in around 15 min and remains visible throughout the whole thoracic stage (Figure 2a) [27].

After deploying the thoracic trocars (Figure 2c), complete exposure of the esophagus can be achieved through the dissection of the visceral pleura and the arch of the Azygos vein, which does not involve any vascular damage and is selectively ligated with hemo-lock clips. The upper dissection of the esophagus extends differently according to the primary tumor level and histology, and involves the preservation of a pleural flap used to suspend the gastric tube at the end. Recurrent laryngeal lymphadenectomy is performed routinely in carinal–supracarinal SCC while on demand in adenocarcinoma. In this context, the inter-cavotracheal and parabronchial stations (including 105 and 106TB R and L) are separately excised.

Inferiorly, the lateral dissection of the esophagus starts from the pleural incision along the Azygos vein course up to the diaphragm and involves the en bloc excision of the pre-aortic soft tissue and lymph node stations (110, 111, 112). In this space, the anatomical proximity between the esophageal body and the TD increases the risk of injuries of the duct and subsequent chyle leaks; therefore, preemptive TD ligation is highly recommended. The combined near-infrared (NIR) and white light vision permit clear TD identification and accurate fluorescence-mediated ligation of the duct at the diaphragmatic and aortic arch levels. The remaining visceral dissection is performed from the inferior pulmonary ligament up to the pulmonary veins and anteriorly to the tracheobronchial tree, with the completion of the mediastinal lymphadenectomy through the dissection of subcarinal stations (107, 109 R and L) en bloc with the esophagus. At the level of the right bronchus, lower and upper esophageal dissection plans join, and the vagus nerve lying on the lateral surface of the esophagus is then sectioned, preserving the tracheobronchial branches.

### 4.4. Anastomotic Technique

The surgical technique for anastomotic fashioning comprises three consecutive stages: (i) the esophageal purse string suture, sectioning, and anvil placement, (ii) intrathoracic transposition and preparation of the gastric conduit, and (iii) circular stapling and anastomosis completion. The successful accomplishment of all procedural stages depends on the entire surgical team’s training and coordination.

**Figure 3:**
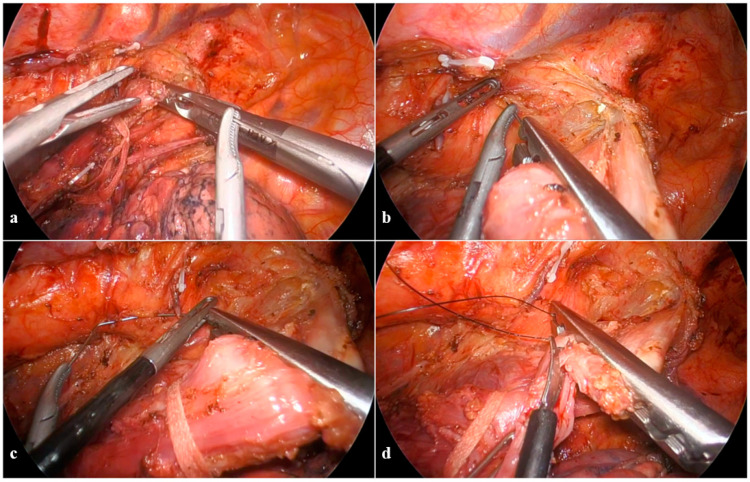
Anastomotic technique: the esophageal purse string suture and sectioning. (**a**) Purse-string instrument grasping the esophagus; (**b**) doubled straight-cutting needle with 2-0 polypropilene suture passing through the purse-string instrument while shielding the aorta; (**c**) the two-hand maneuver of needle extraction; (**d**) separate section of each layer of the esophageal wall.

**Figure 4:**
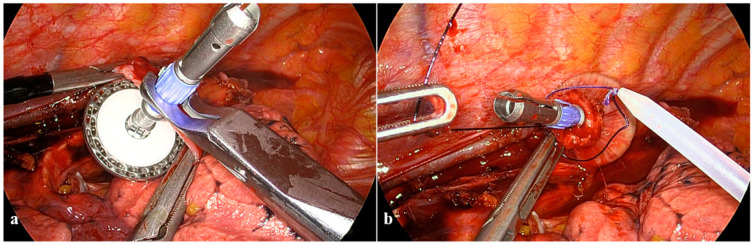
Anastomotic technique: the anvil placement. (**a**) Anvil insertion into the esophageal stump; (**b**) purse-string suture and additional Endoloop™ reinforcement.

In the first stage (i), a dedicated purse-string instrument (Karl Storz) is placed on the esophagus above the carina and places the purse-string suture by progressive insertion of a doubled straight-cutting needle with a 2-0 polypropilene suture (Figure 3a). Given the close proximity between the esophagus and the aortic arch, needles should carefully progress from anterior to posterior through the full esophageal wall thickness; extraction is the most difficult step and should be performed with a two-hand maneuver with the needle-holder in the right hand never leaving the needle tip and bending it, while the grasper in the left hand gently pulls with millimetric steps the needle from its channels (Figure 3b,c). Separate section of each esophageal layer facilitates specimen separation and preserves a robust mucosal rim, which strengthens the anastomosis (Figure 3d). The size of the circular stapler for the esophagogastric anastomosis is chosen upon the esophageal lumen diameter; we normally prefer the size to be as big as possible (normally 28 mm), preferring the PCEEA (Medtronic) for the low profile of the anvil that facilitates insertion into the esophageal stump. The inferior-medial thoracoscopic access is then enlarged for minithoracotomy enforcement (around 5 cm), which is mandatory for anvil insertion, specimen extraction, and gastric conduit preparation. The purse string on the esophagus is tied on the anvil and reinforced with an additional Endoloop™ (Figure 4a,b).

**Figure 5:**
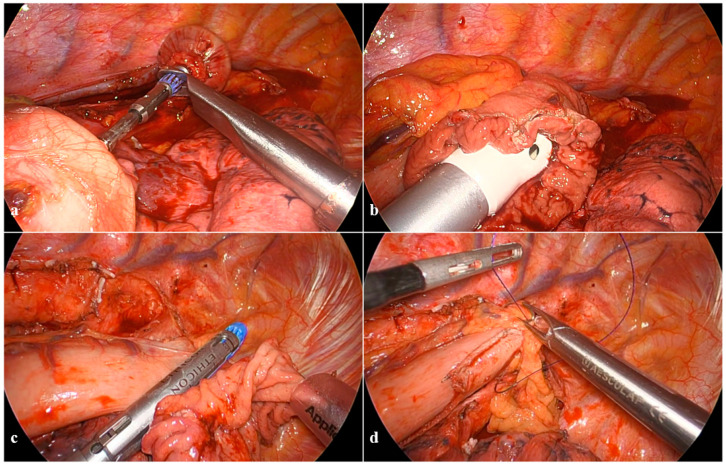
Anastomotic technique: the circular stapling and anastomosis completion. (**a**) Circular stapler assembling with the anvil; (**b**) complete closure of the device; (**c**) full-thickness mechanical suture closing the previous gastrostomy; (**d**) pleural flap and the residual omentum sutured to the greater curvature and the apex of the conduit.

The second stage (ii) involves the careful transposition of the gastric conduit from the abdomen to the chest, applying delicate tractions to prevent either vascular or visceral injuries. A second routine ICG angiography is performed to confirm the gastroepiploic arcade integrity, assess the gastric conduit perfusion, and determine the definitive anastomotic site. The gastric conduit is extracted through the minithoracotomy to facilitate specimen removal and the preparation of the lateral gastric surface at its cranial extremity. This preparation includes establishing the gastric anastomotic side near the distal branches of the gastroepiploic arcade and the intraluminal placement of the circular stapler from the residual gastrostomy after having separated the specimen. Eventually, the circular stapler is deployed, fully unfolding the device’s central rod through the stomach and carefully guiding the gastric conduit to the chest (Figure 5a).

In the third stage (iii), the circular stapler is assembled to the anvil, with both parts gradually approximated until the complete closure of the device with the stapler indicator in the green zone after having checked that the gastric conduit is correctly oriented and the gastroesophageal sides are located at the suture tying area without extraneous tissue over the transection area (Figure 5b). After releasing and removing the device, a linear stapler closes the previous gastrotomy with a full-thickness mechanical suture (Figure 5c). Intraoperative methylene blue testing through the nasogastric tube that is pushed into the conduit rules out potential anastomotic spillage. A short suture involves the pleural flap and the residual omentum along the greater curve and the apex of the conduit (Figure 5d).

Lastly, the transhiatal Jackson–Pratt suction drain is placed near the anastomosis, and a catheter for postoperative analgesia is positioned at the VI intercostal space under thoracoscopic guidance reaching the paravertebral space.

## 5. Discussion

The two-field Ivor Lewis esophagectomy is a major surgical procedure in esophageal cancer surgery, and the hereby-reported surgical technique (MILE-lt) has been fully standardized and represents a key component to minimize postoperative morbidity and mortality. The successful implementation of MILE-lt depends on multiple factors, including the high proficiency of all team members of both the operative room and surgical ward, the infrastructural resource availability combined with the experience of individual centers, and the establishment of multidisciplinary care pathways for esophageal cancer.

The introduction of standardized perioperative clinical protocols, such as multidisciplinary team management or ERAS-based programs, has historically led to a significant optimization of postoperative outcomes [28,29]. In particular, the development of integrative programs combining prehabilitation with enhanced postoperative recovery demonstrated earlier functional restoration, leading to reduced postoperative morbidity, shorter hospital stay, and improved survival and quality of life [30].

In accordance with the literature, the authors of the present study proposed a standardized esophagectomy including a gastric conduit of about 40 mm in width [31]. Although the recent introduction of fluorescent angiography clearly provided immediate and momentary feedback on the gastroepiploic arcade integrity and gastric perfusion during surgery, shaping a wide conduit still represents the most reliable prevention for postoperative ischemia and anastomotic complications. However, optimal dimensions of the gastric conduit are meant to achieve the balance between sufficient blood supply and effective conduit emptying, even though postoperative hemodynamic changes can also significantly interfere [32]. Postoperative hypotensive events contribute to hypoxemia and poor tissue perfusion, with the subsequent risk of ischemic deterioration and anastomotic leakage [25,33]. For this reason and with the purpose of excluding all possible determinants of hypotension, our center moved to the institutional disposition to avoid epidural analgesia in esophageal cancer surgery in favor of the routine placement of a paravertebral analgesia catheter.

Final technical considerations involve comparing MILE-lt with robotic Ivor Lewis esophagectomy. Currently, no evidence has demonstrated the superiority of any specific type of minimally invasive approach regarding either short- or long-term outcomes, while hospital costs appeared to be predominantly loaded by postoperative complications [34]. On the other hand, the reported surgical technique is not limited by the hardware or the financial restrictions of using the robotic platform, increasing the interest in this minimally invasive procedure that does not need specific requirements other than high individual proficiency and the considerable resources that this surgical field usually demands. In these terms, the present description should be considered as a partial component of a much wider perioperative clinical management protocol, where complication treatment appears to stress and burden the most. Thus, only high-volume centers with specialized multidisciplinary teams are more likely to achieve improved complication and survival rates regardless of the surgical approach [35,36].

## 6. Conclusions

The successful implementation of the MILE-lt surgical technique depends on a wide variety of procedural details and tailored expertise. However, despite the accurate description of the strengths and flaws of the present laparo-thoracoscopic approach, beneficial outcomes cannot be achieved regardless of the multidisciplinary context of different professionals practicing in a high-volume setting. According to this article, the total minimally invasive technique combined with the two-field Ivor Lewis esophagectomy within an ERAS-based standardized program may provide patients with improved postoperative recovery and morbidity. The precision and minimally invasive nature of this approach highlight the importance of a dedicated team and resources, working jointly on a comprehensive patient-centered approach to achieving optimal surgical outcomes.

## Data Availability

No new data were created or analyzed in this study. Data sharing is not applicable to this article.

## Supplementary Materials

The following supporting information can be downloaded at https://www.mdpi.com/article/10.3390/cancers16193281/s1, S1: The SUPER Checklist; S2: Surgical instrument equipment. Reference [13] is cited in the supplementary materials.
