# Supplementary material for: Surgical Technique and Implementation of Total Minimally Invasive (Laparo-Thoracoscopic) Ivor Lewis Esophagectomy for Cancer
Source: Cancers (Basel). 2024 Sep 26;16(19):3281. doi: 10.3390/cancers16193281 (PMC11475372; doi:10.3390/cancers16193281)
Supplement: Supplementary file 1 [file cancers-16-03281-s001.zip › Supplementary material/SM - 1. SUPER checklist.docx]

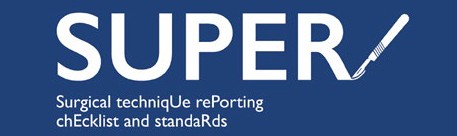
**Surgical techniqUe rePorting chEcklist and standaRds (SUPER)**

| **Section/Topic** | **Item No** | **Recommendation** | **Reported on Page Number/Line Number** | **Reported on Section/Paragraph** |
| --- | --- | --- | --- | --- |
| **Background, Rationale, and Objectives** | | | | |
| Background | 1 | Describe the background of the disease or condition (e.g., its definition, classification, clinical manifestations, epidemiological characteristics, and natural history). | 5 / 3-7 | Introduction |
| Rationale | 2a | Describe the pros and cons of existing treatments for the disease or condition, including currently used single or combined surgical techniques. | 5 / 18-26 | Introduction |
|  | 2b | Explain whether the proposed surgical technique is a novel or modified procedure, including whether any modifications have been made to key devices or materials. If only a conventional surgical technique is used, a brief description should be accompanied by a citation of a source which describes the surgical technique in detail. | 5 / 22-28 | Introduction |
| Objectives | 3 | State what objectives and challenges the proposed surgical technique will address. Introduce what the surgical technique figure and video will cover. | 3 / 16-21  6 / 26-28  7 / 13-15 | Abstract  Introduction  Materials and Methods |
| Classification | 4 | Classify the surgical technique, either by: (i) surgical approach: open, minimally invasive (e.g., thoracoscopic, robotic), or hybrid; or (ii) treatment goal: curative or palliative. | 6 / 26-28 | Introduction |
| Name | 5 | Report the names of all involved surgical techniques in the title or abstract. If the surgical technique is the focus of the paper, also include “surgical technique” in the title. | 1 / 3-4  3 / 13-25 | Title  Abstract |
| **Preoperative Preparations and Requirements** | | | | |
| Setting | 6a | Report information or requirements of the surgical environment (e.g., the name of the hospital, the hospital grade such as tertiary hospital, the degree of cleanliness, and whether the procedure must be performed in an operating theatre). | 7 / 5-13 | Materials and Methods |
|  | 6b | List and provide details of any special surgical equipment, supplies, drugs, or software used (e.g., the manufacturer, product model, quantity, dosage, route, duration, and parameters). | Surgical instrument equipment | Supplementary material |
| Operators | 7 | Provide information about the surgical team personnel, including their role (e.g., surgeon, anesthetist, nurse), learning curve (e.g., the number of cases), and training needed if applicable. | 7 / 10-13 | Materials and Methods |

| Recipients | 8 | Report detailed indications and contraindications.   1. Disease or condition: type, etiology, the location, shape and size of the lesion, etc. 2. Recipients: age, sex, clinical manifestations, disease stage and severity, comorbidities and related complications, surgical history and relevant family history, preoperative tests, pre-intervention, and other factors pertinent to successful practice. | 7 / 18-37 | Materials and Methods |
| --- | --- | --- | --- | --- |
|  | 9 | Provide detailed generic information and preparations.   1. Generic information: de-identified demographic information, symptoms and signs, imaging findings, staging, comorbidities, and relevant therapy history, etc. 2. Preparations: cardiovascular, gastrointestinal and respiratory tract preparation, urinary catheterization, skin preparation, blood product preparation, anesthetic procedure and management, and patient positioning, etc. | 9 / 4-31 | Multidisciplinary complements |
| **Surgical Technique Details** | | | | |
| Surgical approach, key anatomic landmarks, and adjacent structures | 10a | Describe in detail how to establish the surgical approach (e.g., devices and equipment used, the position of the surgeons, anatomic localization, and the incision type, length, size, depth, angle, and number). | 12 / 4-22 | Operative technique |
|  | 10b | Describe the essential anatomic landmarks and adjacent structures, including areas, structures, blood vessels, and nerves, etc. (e.g., “use the Louis angle between the sternal manubrium and the sternal body to find the second costal notch”). | 12 / 4-22 | Operative technique |
| Intraoperative monitoring | 11 | Describe intraoperative monitoring specifically related to the surgical technique (e.g., near-infrared spectroscopy in aortic arch surgery). | 10 / 34-54 | Multidisciplinary complements |
| Step-by-step description | Include all relevant details of each operative step in a step-by-step manner along with both quantitative and qualitative description. | | | |
|  | 12a | Details may include the intraoperative findings, timeline, histomorphology, exposure of vital structures, extent of lymph node dissection, determination of surgical margins, suture pattern (running suture or single stitches; spacing of stitches), anastomosis, knot-tying, specimen handling, and devices/supplies/drugs/blood products used, etc. | 13 / 25-127 | Operative technique |
|  | 12b | Note the operative time. | n.a. | n.a. |
|  | 12c | If a non-conventional maneuver was applied, specify the reason. | n.a. | n.a. |
| Quality and consistency | 13 | Describe tips and skills for ensuring surgical quality and consistency, especially for the key steps and any conditions or variations that require uniform management (if applicable). For example, using standardized training, establishing quality control teams, and organizing multidisciplinary consultations. | 18 / 3-9 | Discussion |

| Safety | 14 | Describe tips and skills for ensuring safety. For example, how to prevent or deal with possible intraoperative complications and emergencies, or when and how to undertake a surgical conversion. | 12 / 18-21 | Operative technique |
| --- | --- | --- | --- | --- |
| Visualization | 15a | Visualize the key steps in a step-by-step and self-explanatory manner. Consider using narrated video(s) and anatomic illustration(s) with designated symbols and illustrated text. | Figure 1-5 | Operative technique |
|  | 15b | The key information in item 12 should be visualized; it can either be presented as a stand-alone figure or embedded in the video(s). | Figure 1-5 | Operative technique |
|  | 15c | Visualization of the key information in items 10, 13, and 14 is encouraged as appropriate. | n.a. | n.a. |
|  | 15d | After peer review, add clips into the video(s) to present the video title, operator name, and operation date at the beginning, and the informed consent and the ethical approval statements at the end. | n.a. | n.a. |
| **Postoperative Considerations and Tasks** | | | | |
| Evaluation | 16a | Define the criteria for success and failure, and evaluate the efficacy or effectiveness of the surgical technique from both the technical aspect and the clinical outcome perspective (e.g., length of stay, improvements in short- and long-term mortality, recurrence, survival time, and patient impairment). | 18 / 10-15 | Discussion |
|  | 16b | When possible, include the perspective of the patient (e.g., symptoms and signs, postoperative pain, and aesthetic results). | 14 / 54-57 | Operative technique |
| Postoperative monitoring | 17 | Describe in detail postoperative monitoring specifically related to the surgical technique (e.g., monitoring indicators, devices, frequency or duration, examination, and nursing required). | 10 / 49-54  10 / 27-31 | Multidisciplinary complements |
| Complication prevention and management | 18 | Report the possible or observed postoperative complications and their prevention and management, especially complications that differ from those related to conventional techniques. | 13 / 28-32  14 / 60-63  14 / 74-78 | Operative technique |
| Follow-up | 19a | Report the details of follow-up visits, including pathway, frequency, duration, and indicators (e.g., pathway- ”telephone follow-up”; frequency-”radiological examinations every 3 months”; duration-”up to 3 years”; indicators-poor outcomes, complications, quality of life, and unexpected events). | n.a. | n.a. |
|  | 19b | If applicable, compare the information in item 19a with those of conventional techniques. | n.a. | n.a. |
| **Summary and Prospect** | | | | |
| Strengths, limitations, and outlook | 20 | Discuss the main strengths and limitations of the surgical technique, and provide detailed suggestions for improvement and future outlooks. | 18 / 3-9  18 / 23-27 | Discussion |
| Impact and cost | 21a | Summarize the key points and take-away lessons of the surgical technique and its impact in the clinical setting and on society (e.g., the economic cost). | 19 / 31-39 | Discussion |
|  | 21b | Consider in context the predominant cost and its potential impact on the implementation and adoption of the surgical technique. | 18 / 5-9  19 / 37-39 | Discussion |

| **Other Information** | | | | |
| --- | --- | --- | --- | --- |
| Conflicts of interest, ethical approval, and informed consent | 22 | (i) Specify any potential conflicts of interest; (ii) include the ethics committee or institutional review board approval (and the number when applicable); and (iii) provide the informed consent for publication. | 19 / 28-39 | Discussion |

1. SUPER website: <https://www.thesuper.org/>

2. Zhang K, Wu J, Su Z, Ma Y, Shi Q, Barchi LC, et al. The SUPER reporting guideline suggested for reporting of surgical technique: explanation and elaboration. Gland Surg 2023;12(6):749-766.

3. Zhang K, Ma Y, Wu J, Shi Q, Barchi L, Scarci M, et al. The SUPER reporting guideline suggested for reporting of surgical technique. Hepatobiliary Surg Nutr 2023 ;12(4):534-544.

4. Zhang K, Ma Y, Shi Q, Wu J, Shen J, He Y, et al. Developing the surgical technique reporting checklist and standards: a study protocol. Gland Surg 2021;10(8): 2591-2599.
