# Supplementary material for: Surgical Technique and Implementation of Total Minimally Invasive (Laparo-Thoracoscopic) Ivor Lewis Esophagectomy for Cancer
Source: Cancers (Basel). 2024 Sep 26;16(19):3281. doi: 10.3390/cancers16193281 (PMC11475372; doi:10.3390/cancers16193281)
Supplement: Supplementary file 1 [file cancers-16-03281-s001.zip › Supplementary material/SM - 2. Surgical instrument equipment.docx]

**Surgical instrument equipment**

| **Abbrv.** | **Description** | **Size** | **Qty.** | **Stage** |
| --- | --- | --- | --- | --- |
| *Trocar* | | | | |
| Port#1 | Hasson trocar – disposable-balloon, bladeless, optical trocar | 5-12 mm | 1 | Abdominal, Thoracic |
| Port#2 | Disposable, bladeless, optical trocar | 5-12 mm | 2 | Abdominal, Thoracic |
| Port#3 | Disposable, bladeless, optical trocar | 5 mm | 2 | Abdominal, Thoracic |
| *Laparoscopic Imaging System* | | | | |
| Camera | 10mm Autoclavable Laparoscope, 30 Degree | 10 mm | 1 | Abdominal, Thoracic |
| Video system | Combined white-light 4K-imaging and real-time fluorescence overlay | - | - | Abdominal, Thoracic |
| Warmer | Sterile hot water warmer for laparoscope lens | - | - | Abdominal, Thoracic |
| ICG#1 | Verdye, Diagnostic Green GmbH, AschheimDornach, Germany | 0.3 mg/kg | | Angiography |
| ICG#2 | Verdye, Diagnostic Green GmbH, AschheimDornach, Germany | 25 mg diluted to 1.25 mg/ml | | Lymphography |
| *Laparoscopic instrumentation* | | | | |
| Forceps#1 | Fenestrated, Johan grasping forceps | 10 mm | 1 | Abdominal, Thoracic |
| Forceps#2 | Fenestrated, Johan grasping forceps | 5 mm | 2 | Abdominal, Thoracic |
| Forceps#3 | Fenestrated, bipolar grasping forceps | 5 mm | 1 | Abdominal, Thoracic |
| Forceps#4 | O'Shaughnessy curved dissecting forceps | 5 mm | 1 | Abdominal, Thoracic |
| Forceps#5 | O'Shaughnessy curved dissecting forceps | 10 mm | 1 | Abdominal, Thoracic |
| Forceps#6 | Purse-string suture forcerps | 10 mm | 1 | Thoracic |
| Forceps#7 | Anvil grasper | 10 mm | 1 | Thoracic |
| L-hook | Laparoscopic electrode – unipolar dissector | 5 mm | 1 | Abdominal, Thoracic |
| Energy device | Cutting and sealing energy device (ultrasonic and advanced bipolar energy) | 5 mm | 1 | Abdominal, Thoracic |
| Needle holder | Needle holder | 5 mm | 1 | Abdominal, Thoracic |
| Suction | Irrigation/suction set | 5 mm | 1 | Abdominal, Thoracic |
| Ligation#1 | Titanium clip applier (medium reload) | 10 mm | 1 | Abdominal, Thoracic |
| Ligation#2 | Hem-o-lok clip applier (large/extra-large clip size) | 10 mm | 1 | Thoracic |
| Scissor | Laparoscopic scissor | 5 mm | 1 | Abdominal, Thoracic |
| *Stapling system* | | | | |
| Stapler#1 | Linear stapler (articulating and straight) – Thick tissue (3.8-1.8 mm) | 45 mm | 4-5 reloads | Abdominal |
| Stapler#2 | Linear stapler (articulating and straight) – Medium tissue (3.6-1.5 mm) | 45 mm | 1-2 reloads | Thoracic |
| Stapler#3 | Circular stapler - matching to the diameter of the esophageal lumen | 25-28-31 mm | 1 | Thoracic |
| Stapler#4 | Skin stapler | - | 1 | Abdominal, Thoracic |
| *Other* |  |  |  |  |
| Wound#1 | Berci fascial closure device | - | 1 | Abdominal |
| Wound#2 | Alexis wound protector/retractor | small | 1 | Thoracic |
| Wound#3 | Finocchietto rib spreader | small | 1 | Thoracic |
| Wound#4 | Farabeuf retractors | 12 cm | 2 | Abdominal, Thoracic |
| Endobag | Laparoscopic endo pocket with memory wire | 120 ml | 1 | Thoracic |
| Jejunostomy | Kangaroo feeding jejunostomy kit | - | 1 | Abdominal |
